# Vascular endothelial growth factor receptor-2 and its association with tumor immune regulatory gene expression in hepatocellular carcinoma

**DOI:** 10.18632/aging.104119

**Published:** 2020-11-20

**Authors:** Ze-Long Liu, Ling-Ling Zhu, Jing-Hua Liu, Zhang-Ya Pu, Zhi-Ping Ruan, Jiang Chen

**Affiliations:** 1 Division of Interventional Ultrasound, The First Affiliated Hospital of Sun Yat-sen University, Guangzhou, Guangdong Province, China; 2 Lung Cancer Center, West China Hospital of Sichuan University, Chengdu, Sichuan Province, China; 3 Department of Hepatobiliary Surgery and Professor Cai’s Laboratory, Linyi People's Hospital, Linyi, Shandong Province, China; 4 Department of General Surgery, Sir Run Run Shaw Hospital, Zhejiang University, Hangzhou, Zhejiang Province, China; 5 Department of Radiation Oncology, Massachusetts General Hospital, Harvard Medical School, Boston, MA 02115, USA

**Keywords:** cancer, immunotherapy

## Abstract

Anti-vascular endothelial growth factor (anti-VEGF) drugs have long been the only first-line treatment for advanced or unresectable hepatocellular carcinoma (HCC). Recently, the combination of bevacizumab (an anti-VEGF drug) and atezolizumab (an immune checkpoint blockade, ICB) has been proven to have superior efficacy over sorafenib. However, the complex association between VEGF signaling pathway and tumor immune microenvironment is still largely unknown. Here, we analyzed the RNA sequencing and clinical data of 365 HCC patients obtained from The Cancer Genome Atlas to investigate the potential correlation between VEGF signaling pathway and tumor immune microenvironment, including immune cell infiltration, 66 immune markers, genomic instability, and immune-related pathways. Our study revealed that VEGF signaling pathway score was positively correlated with immune cell infiltration and the expression profile of 66 immune markers. Enrichment analysis indicated that genes differentially expressed between two VEGF score subtypes were enriched in many immune-related Gene Ontology terms. Most importantly, both VEGF signaling pathway and activated CD8+ T cells were positively correlated with prognosis. Our findings suggest the co-activation of VEGF signaling pathway and tumor immune microenvironment in HCC patients, indicating the underlining mechanism of combination therapy including anti-VEGF drugs and ICBs.

## INTRODUCTION

Hepatocellular carcinoma (HCC) is the fourth leading cause of cancer-related death worldwide [1]. Surgical resection has been adopted as the standard treatment for most patients with early-stage HCC [2, 3]. However, up to 70% of patients experience tumor recurrence within five years after surgery [2, 3]. Therefore, the biggest challenge in the clinical management of HCC is to prevent tumor recurrence and to improve the prognosis of advanced or unresectable disease.

Angiogenesis, the formation of new blood vessels, is crucial in tumor development, growth, and metastasis because blood vessels carry nutrients and facilitate the spread of tumor cells. Vascular endothelial growth factor (VEGF) signaling is important in angiogenesis and has been identified as a therapeutic target in diverse cancer types, including HCC [4]. Among the VEGF receptors, VEGF-receptor 2 (VEGFR2) is now considered the main receptor, and VEGFR2 activation promotes endothelial cell mitogenesis and vascular permeability [4]. Anti-VEGF monotherapy has been the only standard treatment for advanced or unresectable HCC over the past decade after sorafenib was approved by the US Food and Drug Administration (FDA) [5]. After sorafenib, lenvatinib was approved as a first-line treatment, while several other anti-VEGF drugs, including regorafenib, cabozantinib, and ramucirumab, were approved as second-line treatments [6–9]. Sorafenib provided longer overall survival (OS) compared with a placebo and lenvatinib was non-inferior to sorafenib [5, 6]. Despite this, patients with advanced HCC continue to have unsatisfactory clinical outcomes, with an OS of approximately one year [5–9]. To improve patient prognosis, there is an urgent medical need for novel treatments or improved clinical benefits of anti-VEGF drugs.

Over the last decade, cancer immunotherapy such as immune checkpoint blockade (ICB) therapy, has made tremendous breakthroughs in a variety of malignancies. However, only a small subset of HCC patients respond to ICB monotherapy [10, 11]. The major hurdles in achieving satisfactory benefits are high inter- and intra-tumor heterogeneity, various immune microenvironment, lack of predictive markers, and insufficient efficacy of monotherapy. The IMbrave 150 study was a phase 3 clinical trial that compared the efficacy of atezolizumab (ICB) plus bevacizumab (anti-VEGF drug) versus sorafenib as a first-line treatment for patients with unresectable HCC [12]. Recently, the results of this study demonstrated the impressive clinical benefits of the combination therapy of atezolizumab and bevacizumab, which resulted in a significantly prolonged OS [12]. Based on this, the US FDA has approved atezolizumab plus bevacizumab as a novel first-line treatment for advanced or unresectable HCC. In another phase 3 trial, LEAP-002, early clinical data has also indicated promising efficacy for the combination of lenvatinib and pembrolizumab (ICB) [13]. However, the underlying mechanisms of the combination of anti-VEGF drugs and ICBs have not yet been elucidated. Dual PD-1 and VEGFR2 blockade exhibited a synergistic antitumor effect in mouse models of HCC by promoting vascular normalization and enhancing antitumor immune responses [14]. Although the VEGF signaling pathway contributes to immunosuppressive tumor microenvironment, the association between VEGF signaling pathway and immune-related genes is poorly understood [15]. Recently, the expression profile of immune checkpoint genes was shown to be related to the prognosis of HCC patients [16]. Of note, NRP1, an immune checkpoint gene that is also involved in VEGF-VEGFR2 signaling, was found to be an important prognostic factor in HCC [16].

Therefore, we aimed to investigate the association between VEGF signaling pathway, tumor immune microenvironment, and the prognosis of HCC patients using data obtained from The Cancer Genome Atlas (TCGA; n = 365) in this study. It is important to gain a more comprehensive understanding of the field to improve the clinical management of HCC patients, especially for decision making regarding anti-VEGF treatments, ICB therapies, and their combination in patients with advanced HCC.

## RESULTS

### Immune cell infiltration patterns associated with VEGF score subtypes

RNA sequencing and clinical data of 371 HCC patients were downloaded from the TCGA database, among which six were excluded due to incomplete follow-up information. Based on calculated enrichment scores of the VEGF signaling pathway, patients were divided into two VEGF subtypes: the high score subtype with scores in the top one-third (n = 122) and the low score subtype with scores in the bottom two-thirds (n = 243).

**Figure 1:**
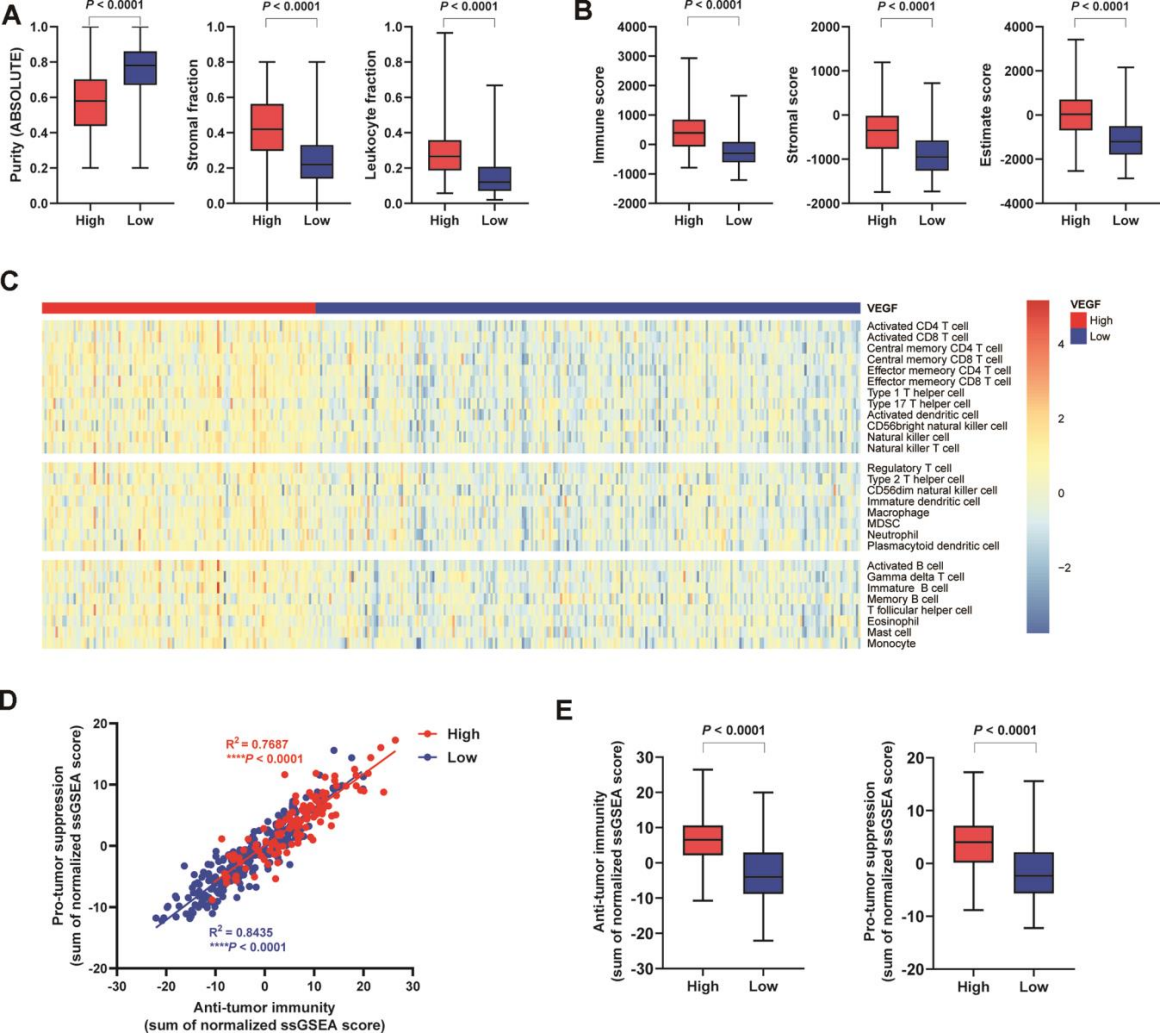
**Immune cell infiltration patterns of the two VEGF score subtypes.** The high VEGF score subtype showed lower tumor purity, higher stroma fraction, and higher leukocyte fraction (**A**). Higher immune score, stromal score, and Estimate score based on the ESTIMATE algorithm were observed in the high VEGF score subtype (**B**). The high VEGF score subtype had relatively higher immune cell infiltration, including cells contributing to both anti-tumor immunity and pro-tumor suppression (**C**). A positive association between these two categories of immune cells in the local tumor microenvironment was observed in both VEGF score subtypes (**D**). The high VEGF score subtype showed both higher anti-tumor immunity and pro-tumor suppression (**E**).

Based on the published data from the TCGA group, we compared the tumor purity and the stromal fraction between the high and the low VEGF score subtype. This revealed a significantly lower tumor purity and higher stromal fraction in the high VEGF score subtype (*P* < 0.0001) (Figure 1A). More importantly, the high VEGF score subtype exhibited a significantly higher leukocyte fraction (*P* < 0.0001) (Figure 1A). Based on the ESTIMATE results, we found that the high VEGF score subtype had a significantly higher immune score, stromal score, and Estimate score (all with *P* < 0.0001) (Figure 1B). Infiltration of 28 immune cell types was estimated for each by using ssGSEA scores. scores. Compared with the low VEGF score subtype, the high VEGF score subtype had relatively higher immune cell infiltration, including cells contributing to anti-tumor immunity (e.g., activated CD8+ T cells, type 1 T helper cells, and natural killer cells) and cells contributing to pro-tumor suppression (e.g., regulatory T cells, immature dendritic cells, and myeloid-derived suppressor cells, MDSCs) (Figure 1C). For each VEGF score subtype, Spearman’s correlation test suggested a positive association between these two categories of immune cells in the local tumor microenvironment (Figure 1D). The high VEGF score group exhibited both higher anti-tumor immunity and pro-tumor suppression (Figure 1E).

**Figure 2:**
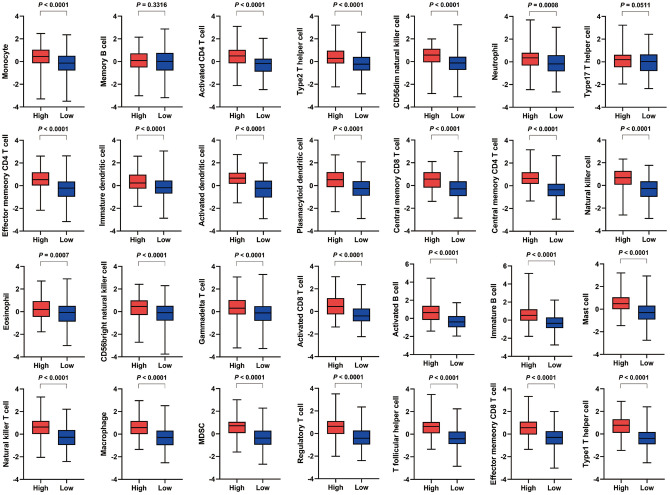
**Comparison of the infiltration of 28 immune cell types between the two VEGF score subtypes.** The high VEGF score subtype exhibited significantly higher infiltration of 26 immune cell types (except for memory B cells and type17 T helper cells).

For specific immune cell types, the high VEGF score subtype had significantly higher infiltration of 26 immune cell types (except for memory B cells and type17 T helper cells) (Figure 2). Spearman’s correlation tests also indicated positive correlations between VEGF signaling pathway ssGSEA scores and the ssGSEA scores of 28 immune cell types (Supplementary Figure 1). Although the absolute levels of immune cells varied between the two VEGF score subtypes, the relative cell fractions of most immune cells estimated by the CIBERSORT algorithm appeared to be similar (Supplementary Figure 2A, 2B). Notably, higher fractions of M1 macrophages, M0 macrophages, and gamma delta T cells were observed in the high VEGF score subtype (Supplementary Figure 2A, 2C).

### Immune-regulatory gene expression profiles associated with VEGF score subtypes

**Figure 3:**
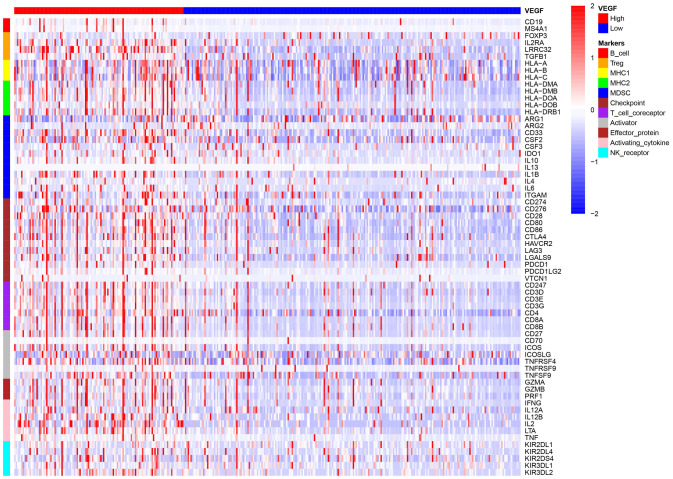
**The high VEGF score subtype showed a higher expression of 66 immune markers associated with immune stimulation or suppression.**

**Figure 4:**
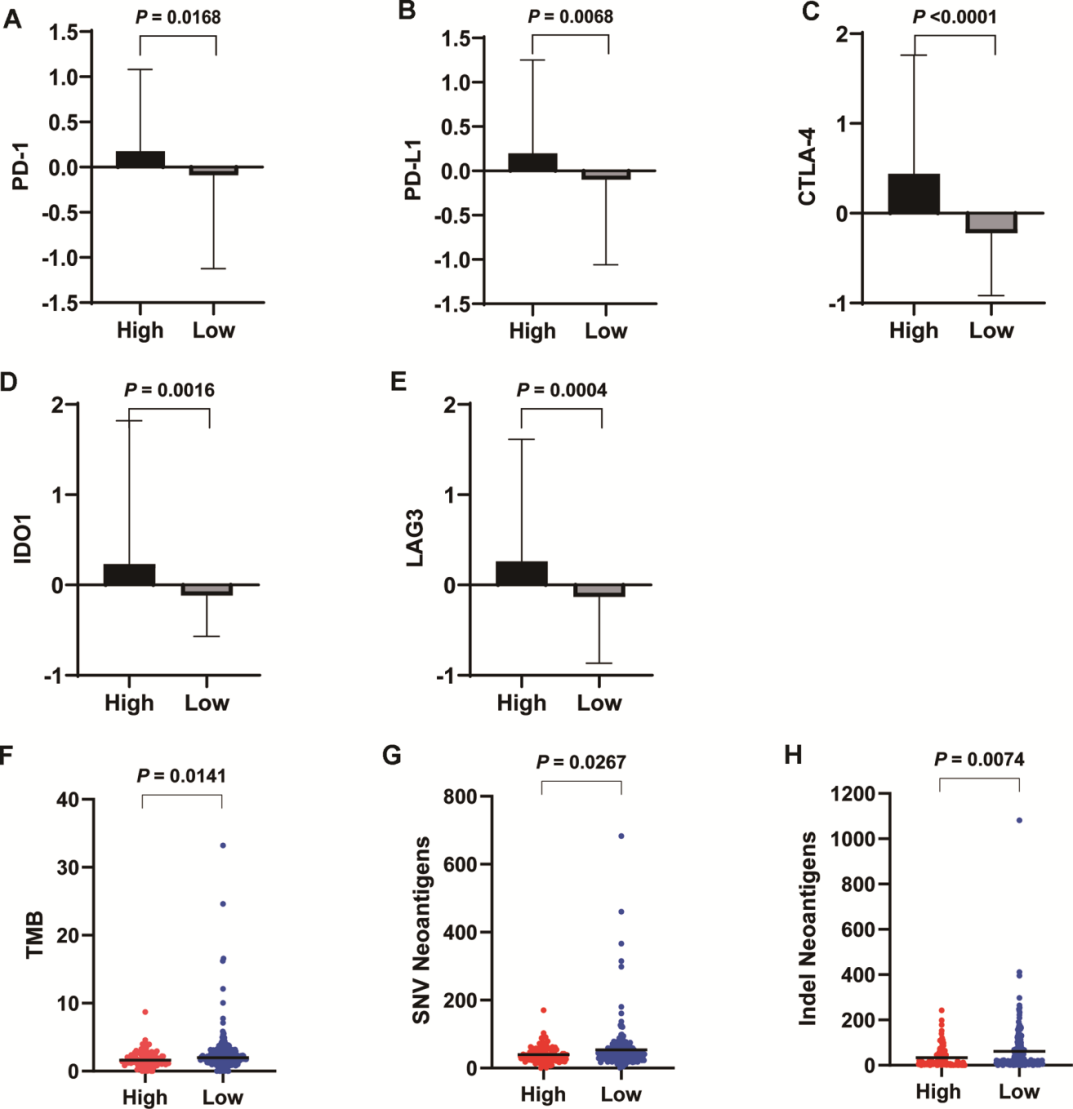
****Expression levels of important checkpoint molecules including PD-1 (**A**), PD-L1 (**B**), CTLA-4 (**C**), IDO1 (**D**), and LAG3 (**E**) were all significantly higher in the high VEGF score subtype. Significantly lower TMB (**F**), SNV neoantigen number (**G**), and indel neoantigen number (**H**) were observed in the high VEGF score subtype.

The high VEGF score subtype exhibited higher expression of 66 immune markers associated with immune stimulation or suppression (Figure 3). The correlations between the 66 immune marker expression scores in all patients, patients of the high VEGF score subtype, and patients of the low VEGF score subtypes were investigated by Spearman’s correlation tests and visualized in Supplementary Figures 3–5, respectively. We then focused on several important checkpoint molecules including PD-1, PD-L1, CTLA-4, IDO1, and LAG3, and found that the expression levels of these molecules were all significantly higher in the high VEGF score subtype (Figure 4A–4E).

To investigate the genomic changes associated with each VEGF score subtype, we compared the TMB, single-nucleotide variant (SNV) neoantigen number, and indel neoantigen number between the two VEGF score subtypes. We found significantly lower TMB, SNV neoantigen number, and indel neoantigen number in the high VEGF score subtype than in the low VEGF score subtype (Figure 4F–4H), which indicated relatively lower genome instability in the high VEGF score subtype.

### DEGs between VEGF score subtypes were enriched in immune-related GO terms

**Figure 5:**
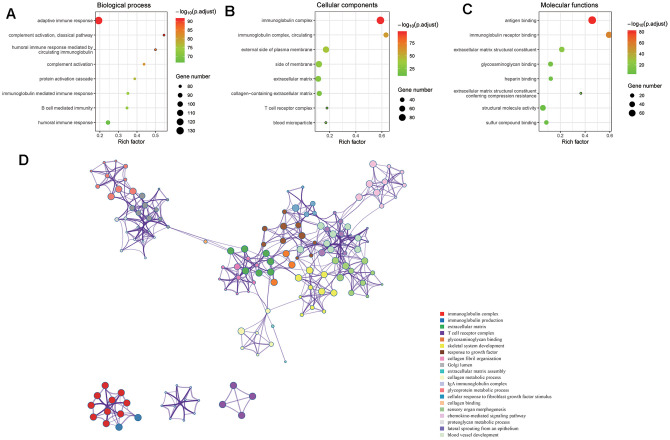
**DEGs between the two VEGF score subtypes enriched in immune-related GO terms.** GO pathway enrichment analysis revealed that immune-related GO terms ranked top in biological process (**A**), cellular components (**B**), and molecular functions (**C**). The network of the top 20 GO summary terms revealed the relationships between immune-related GO terms, angiogenesis-related GO terms, and stroma related GO terms (**D**).

In all, 495 DEGs between the two VEGF score subtypes were identified, among which 456 were upregulated, and 39 were downregulated in the high VEGF score subtype. GO pathway enrichment analysis revealed the following top GO terms: adaptive immune response, complement activation (classical pathway), humoral immune response mediated by circulating immunoglobulin in biological process (Figure 5A); immunoglobulin complex, immunoglobulin complex (circulating), external side of plasma membrane in cellular components (Figure 5B); antigen binding, immunoglobulin receptor binding, extracellular matrix structural constituent in molecular functions (Figure 5C). Moreover, we constructed a network of the top 20 GO summary terms via the Metascape website, which revealed the relationships between immune-related GO terms, angiogenesis-related GO terms, and stroma related GO terms (Figure 5D).

**Figure 6:**
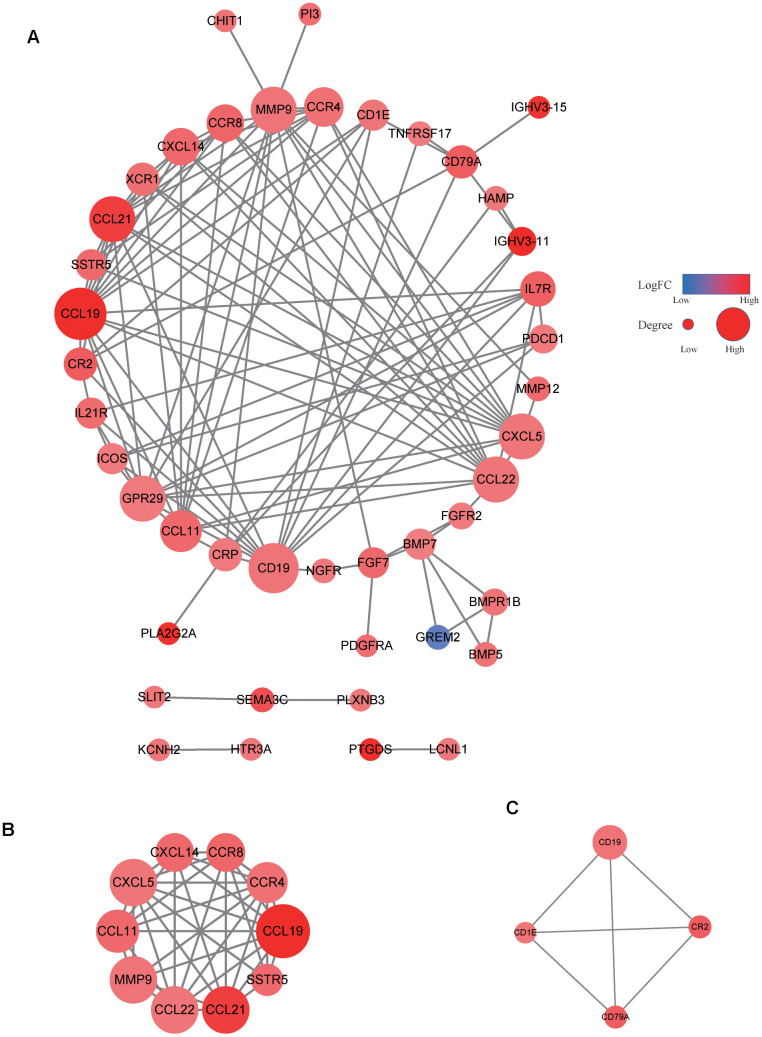
**PPI network of immune-related genes.** The PPI network consisted of 44 nodes and 112 edges (**A**). Two important modules were observed via the MCODE algorithm (**B**, **C**).

Among the 495 DGEs, 172 were immune-related genes according to the data obtained from ImmPort. To further investigate these immune-related genes, a PPI network consisting of 44 nodes and 112 edges was constructed using the STRING database and Cytoscape v3.6.1 (Figure 6A). CCL19 exhibited the highest connectivity degree (15) in the PPI network. Analysis via the MCODE algorithm revealed two important modules with MCODE scores of 8.444 (10 nodes and 38 edges) and 4 (4 nodes and 6 edges) (Figure 6B, 6C). These two top modules included 10 genes (including CCL11, CCL19, CCL21, CCL22, CCR4, CCR8, CXCL5, CXCL14, MMP9, and SSTR5) and four genes (including CD1E, CD19, CD79A, and CR2), respectively (Figure 6B, 6C).

### High VEGF score subtype and high levels of activated CD8+ T cells predict favorable prognosis

**Figure 7:**
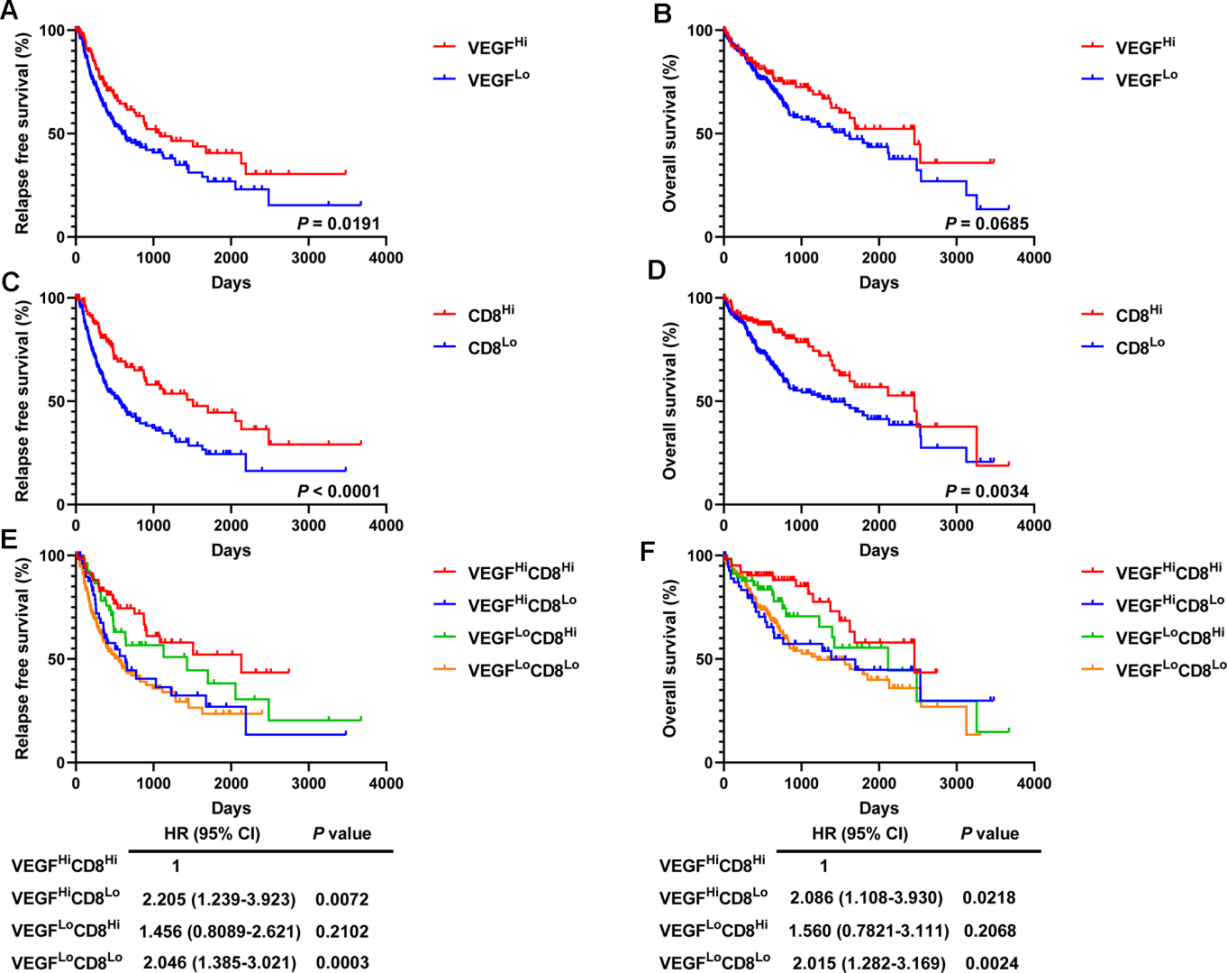
**Survival analysis of 365 HCC patients.** High VEGF score subtype (**A**, **B**) and high activated CD8+ T cells (**C**, **D**) predicted favorable prognosis. Improved RFS and OS were observed in the VEGFHiCD8Hi group compared to the VEGFHiCD8Lo group and the VEGFLoCD8Lo group, but not the VEGFLoCD8Hi group (**E**, **F**).

Given the prognostic value of CD8+ T cells in a variety of solid tumors, we then divided the 365 HCC patients into a high activated CD8+ T cell group (CD8Hi, n = 122) and a low activated CD8+ T cell group (CD8Lo, n = 243) based on ssGSEA scores. Compared with the low VEGF score subtype, the high VEGF score subtype had a significantly longer recurrence free survival (RFS, *P* = 0.0191) and the tendency of a longer OS (*P* = 0.0685) (Figure 7A, 7B). We also observed significantly better RFS and OS in the CD8Hi group when compared with the CD8Lo group (*P* < 0.0001 and *P* = 0.0034, respectively) (Figure 7C, 7D). We then categorized the 365 patients into four groups (VEGFHiCD8Hi, VEGFHiCD8Lo, VEGFLoCD8Hi, and VEGFLoCD8Lo) based on the ssGSEA scores of the VEGF signaling pathway and activated CD8+ T cells. Upon comparing RFS and OS between the groups, both suggested better prognosis in the VEGFHiCD8Hi group compared with the VEGFHiCD8Lo group and the VEGFLoCD8Lo group, but not the VEGFLoCD8Hi group (Figure 7E, 7F).

## DISCUSSION

Cancer immunotherapies, particularly ICBs targeting the PD-1/PD-L1 and CTLA-4 immune checkpoints, have made great success in a variety of malignancies over the past decade and have revolutionized treatment strategies for many cancer patients. However, for HCC, the response rate and prognostic benefit of ICBs alone appear to be extremely limited [10, 11]. Recent evidence has impressively demonstrated that combination therapy of anti-VEGF drugs and ICBs provides promising efficacy in HCC patients [12, 13]. The success of such combination therapy requires a better understanding of the complex associations between the VEGF signaling pathway and the tumor immune microenvironment.

Pathologic angiogenesis plays a critical role in tumor development and progression. There is now much evidence demonstrating that the VEGF/VEGFR2 signaling pathway is one of the most important pathways regulating angiogenesis. Activation of the VEGF signaling pathway promotes endothelial cell proliferation and vascular permeability, which can facilitate immune cell infiltration [8]. Additionally, by altering the adhesion molecules expressed on immune cells (e.g., integrin ligands intercellular adhesion molecule 1) and endothelial cells (e.g., vascular cell adhesion protein 1), VEGF can control the trafficking of immune cells to tumors [15]. However, VEGF has also been demonstrated to induce immunosuppression through several mechanisms, including inhibiting the trafficking, proliferation, and effector function of cytotoxic T cells and the maturation and antigen presentation of dendritic cells, promoting the recruitment and proliferation of immunosuppressive cells, and leading to a hypoxic and low-pH immunosuppressive microenvironment [15]. In our study, using RNA sequencing data from TCGA database, we found that there were more infiltrated immune cells, including both immune-active cells and immune-suppressive cells, in the high VEGF score subtype, which supports previous theories. Moreover, positive correlations between VEGF scores and immune cell scores were observed for most of the 28 immune cell types. These findings implied that both immune-active cells and immune-suppressive cells might enter the tumor microenvironment when VEGF-related molecules are upregulated. This also indicates the potential for feedback from the recruitment and infiltration of immune-active cells to facilitate immune suppression. Notably, we found a lower fraction of M1 macrophages and a higher fraction of M2 macrophages in the low VEGF score subtype. High infiltration of M2 macrophages in the tumor stroma has been reported to be associated with a down-regulating antitumor immune response [30]. In contrast, the high VEGF score subtype exhibited a higher fraction of M1 macrophages, indicating a potentially more responsive tumor status to ICBs. This result was inconsistent with the previous findings that VEGF could contribute to macrophage polarization from M1 to M2 [15]. Given that the total amount of macrophages was also higher in the high VEGF score subtype and the spatial organization of macrophages was unclear, the relationship between VEGF and macrophages requires further investigation. Additionally, the fraction of gamma delta T cells was relatively higher in the high VEGF score subtype. Gamma delta T cells have been found to possess cytotoxic antitumor activity and insufficient levels of functional gamma delta T cells is associated with tumor recurrence and poor prognosis [31–33]. Given that the role of gamma delta T cells in HCC is poorly understood, further studies are needed to validate our finding.

Our data revealed that the expression levels of 66 immune markers, both immune stimulation-related genes and immune suppression-related genes, were higher in the high VEGF score subtype. Notably, immune checkpoint genes, including PD-1, PD-L1, CTLA-4, IDO1, and LAG3, also exhibited significantly higher expression levels in the high VEGF score subtype. This indicated that the tumor was more likely to be responsive to ICB treatments in high VEGF score subtype. Additionally, the high VEGF score subtype had both lower TMB and neoantigen numbers compared to the low VEGF score subtype, indicating lower genetic instability. High immune infiltration is thought to lower genetic instability by eliminating tumor subclones and inhibiting tumor evolution [34]. On the contrary, insufficient immune infiltration might allow immune escape, leading to tumor evolution and increased genomic instability [34]. Together, these findings suggest that the VEGF high score subtype is more immune inflamed and may be more likely to benefit from ICB treatments. In agreement with previous findings, numerous DEGs between the two VEGF score subtypes were immune-related genes (172 of 495). To comprehensively explore the differences in gene expression profiles, we conducted GO pathway enrichment analysis, which indicated that many immune-related GO terms were ranked top in biological process, cellular components, and molecular functions. Other top GO terms were primarily associated with stromal components (e.g., extracellular matrix, extracellular matrix structural constituent), which was also consistent with the high stromal score in the high VEGF score subtype. The network of the top 20 summary GO terms further indicated the interplay between the immune microenvironment, the development of blood vessels, and the stroma. Additional PPI analysis focused on the immune-related genes revealed two important modules that included 10 genes (including CCL11, CCL19, CCL21, CCL22, CCR4, CCR8, CXCL5, CXCL14, MMP9, and SSTR5) and four genes (including CD1E, CD19, CD79A, and CR2), respectively. The cytokines in the first module primarily display chemotactic activities for miscellaneous immune cells, which explain the higher immune infiltration in the high VEGF score subtype. Genes in the second module were associated with encoding important components of B cells, which are important in humoral immunity.

Although both immune active factors and immune suppressive factors were present in the local tumor microenvironment, the former appeared to be the dominating components. Our survival analysis also supported the findings mentioned above and indicated a better prognosis (especially RFS) for the high VEGF score subtype. Another possible explanation is that HCC patients of the high VEGF score subtype might respond better to widely used anti-VEGF therapies. Given the prognostic value of CD8+ T cells in various malignancies, we next focused on them. Activated CD8+ T cells, in agreement with previous literature, predicted favorable clinical outcomes in HCC patients [35–38]. We also observed that the survival curves of the VEGFLoCD8Lo group and the VEGFHiCD8Lo group were similar and lower than the other two groups (the VEGFHiCD8Hi group and the VEGFLoCD8Higroup), suggesting an important role for immune infiltration accompanying angiogenesis in eliminating the tumor.

One of the biggest limitations of our study is that there is no information regarding the spatial organization of immune cells in the tumors. As previously demonstrated, center tumor immune cells, but not invasive margin immune cells, primarily contribute to tumor elimination and favorable prognosis [39, 40]. Additionally, although we considered the whole VEGF/VEGFR2 pathway rather than single molecules to better represent angiogenesis, vessel development in tumors is indeed very complicated and could be affected by many other factors. Finally, we demonstrated the correlation between VEGF signaling and tumor immune microenvironment, but not a causal relationship. Given that our findings are based on bioinformatics analysis, further laboratory experiments are needed to validate these results.

## CONCLUSIONS

In conclusion, our study found the potential co-activation of VEGF signaling pathway and tumor immune microenvironment, indicating a potential benefit for combination therapy including anti-VEGF and ICBs in HCC patients.

## MATERIALS AND METHODS

### Data sources

The level 3 RNA sequencing and clinical data of HCC patients were retrieved from TCGA data portal (https://portal.gdc.cancer.gov).

### Gene signatures and single-sample gene set enrichment analysis (ssGSEA) scores

The enrichment score of the VEGF signaling pathway (the KEGG_VEGF_SIGNALING_PATHWAY, Supplementary Figure 6), was calculated using ssGSEA. The KEGG_VEGF_SIGNALING_PATHWAY only focused on the VEGF/VEGFR2 axis. The gene set representing the KEGG_VEGF_SIGNALING_PATHWAY was downloaded from the GSEA Molecular Signatures Database (MSigDB) v7.1 (https://www.gsea-msigdb.org/gsea/downloads.jsp) and includes a total of 76 genes [17, 18]. Data on tumor purity, stromal fraction, and leukocyte fraction were obtained from a previously published study from the TCGA group [19]. For the quantification of infiltrated immune cells, we utilized a previously published calculation method based on the expression profiles of 782 genes from 28 immune cell types [20]. The degree of immune infiltration was estimated by the ssGSEA scores through the Gene Set Variation Analysis (GSVA) package in R v3.6.2 [21]. We obtained immune scores, stromal scores, and Estimate scores, which were calculated by using the ESTIMATE algorithm, for TCGA cohorts from the official data portal (https://bioinformatics.mdanderson.org/estimate/) [22]. The CIBERSORT algorithm (https://cibersort.stanford.edu) was used to determine the relative abundances of 22 immune cell types in the tumor microenvironment [23].

### Immune signature and immune markers

To investigate the expression of immune markers, we calculated expression scores using a panel of 66 immune markers that are associated with immune stimulation or suppression in the tumor microenvironment [24]. To determine if VEGF signaling pathway subtypes are associated with genomic changes, we downloaded tumor mutation burden (TMB) and neoantigen data from two previously published studies from the TCGA group [19, 25].

### Identification of differentially expressed genes (DEGs) and Gene Ontology (GO) pathway enrichment analyses

RNA sequencing data was analyzed using the limma package in R v3.6.2 to identify DEGs with cutoff values of |log2FC| ≥ 1.5 and FDR < 0.05 [26]. GO pathway enrichment analyses of DEGs were performed using the Metascape website (http://metascape.org) [27].

### Functional enrichment of protein-protein interaction (PPI) network and module analysis of immune-related DEGs

Immune-related genes were extracted from the identified DEGs, based on the immune-related gene list downloaded from the Immunology Database and Analysis Portal (ImmPort) (https://immport.niaid.nih.gov). We utilized the STRING database to construct and download PPIs among the immune-related DEGs [28]. Cytoscape v3.6.1 was used to reconstruct the PPI network and calculate the connectivity degree of each node [29]. Hub clusters in the network were detected and visualized using the molecular complex detection (MCODE) algorithm.

### Statistical analysis

Continuous parameters were compared using the Student’s t test. The correlations between the VEGF signaling pathway ssGSEA scores and the ssGSEA scores across 28 immune cell types were determined by Spearman’s correlation test. The correlation between the immune marker expression scores was determined by Spearman’s correlation test and was visualized using the corrplot package in R v3.6.2. To visualize and compare immune cell infiltration patterns and immune signatures across different VEGF signaling pathway subtypes, we generated heatmaps using the pheatmap package in R v3.6.2. Bubble plots were plotted using the ggplot2 package in R v3.6.2. For survival analysis, Kaplan–Meier curves were plotted and compared using the Cox proportional-hazards regression model. In all analyses, a *P* value of a two-sided test less than 0.05 was considered to be statistically significant. All statistical analyses were performed with GraphPad Prism v8.3.0 and R v3.6.2.

## Supplementary Material

Supplementary Figures
