# Supplementary material for: Vascular endothelial growth factor receptor-2 and its association with tumor immune regulatory gene expression in hepatocellular carcinoma
Source: Aging (Albany NY). 2020 Nov 20;12(24):25172–88. doi: 10.18632/aging.104119 (PMC7803564; doi:10.18632/aging.104119)
Supplement: Supplementary Figures [file aging-12-104119-s001.pdf]

SUPPLEMENTARY FIGURES

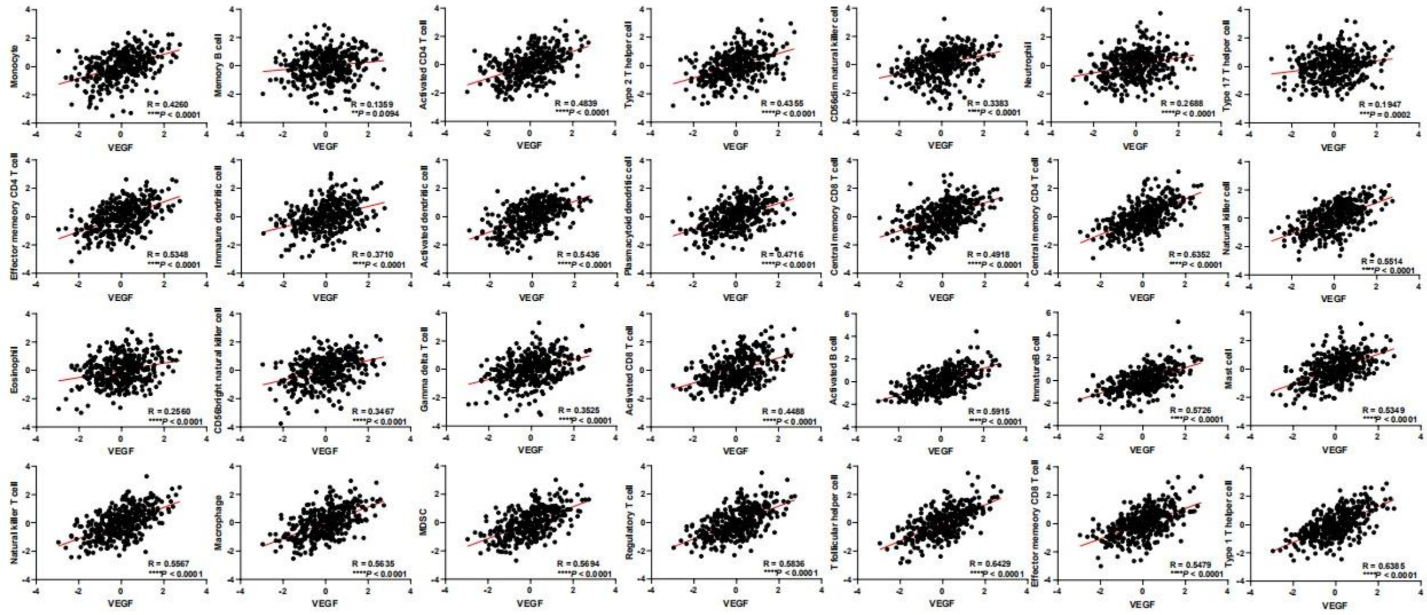

Supplementary Figure 1. Spearman's correlation tests indicated positive correlations between the ssGSEA score of the VEGF signaling pathway and the ssGSEA scores of 28 immune cell types.

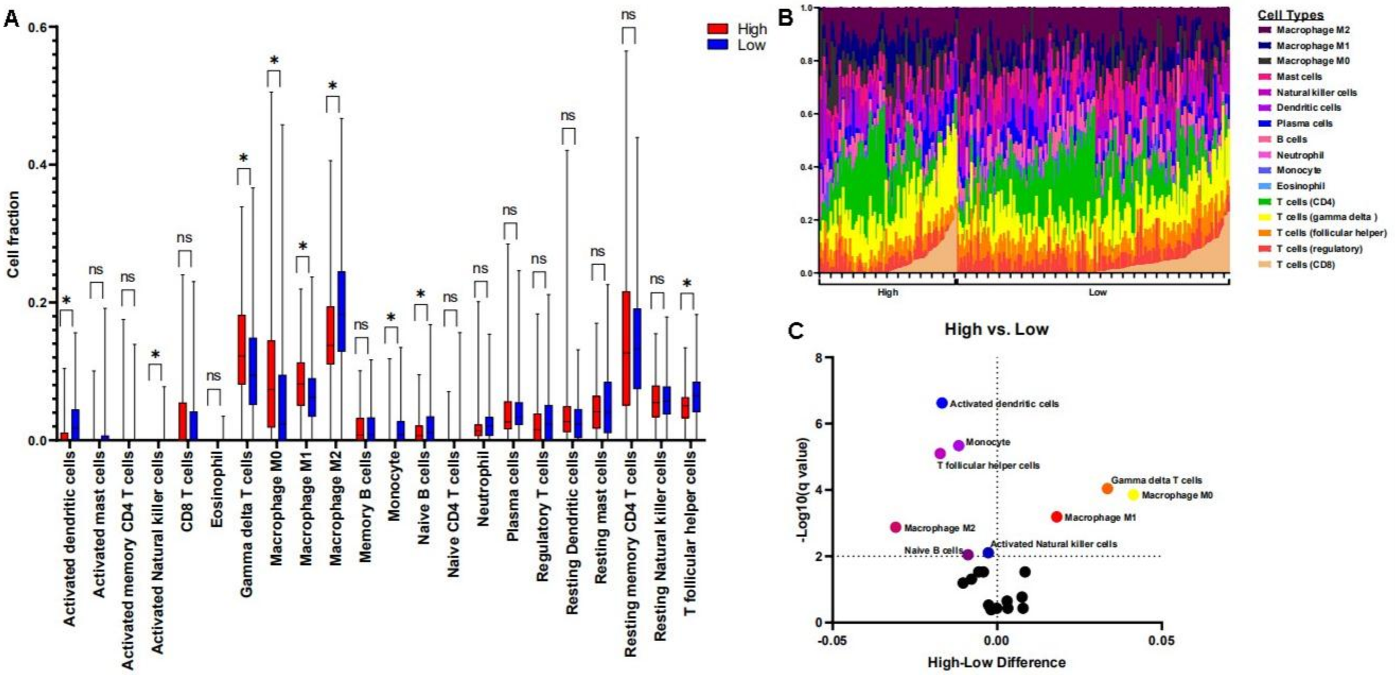

Supplementary Figure 2. The relative cell fractions of most immune cells estimated by the CIBERSORT algorithm appeared to be similar (**A**, **B**). Higher fractions of M1 macrophages, M0 macrophages, and gamma delta T cells were observed in the high VEGF score subtype (**A** and **C**).

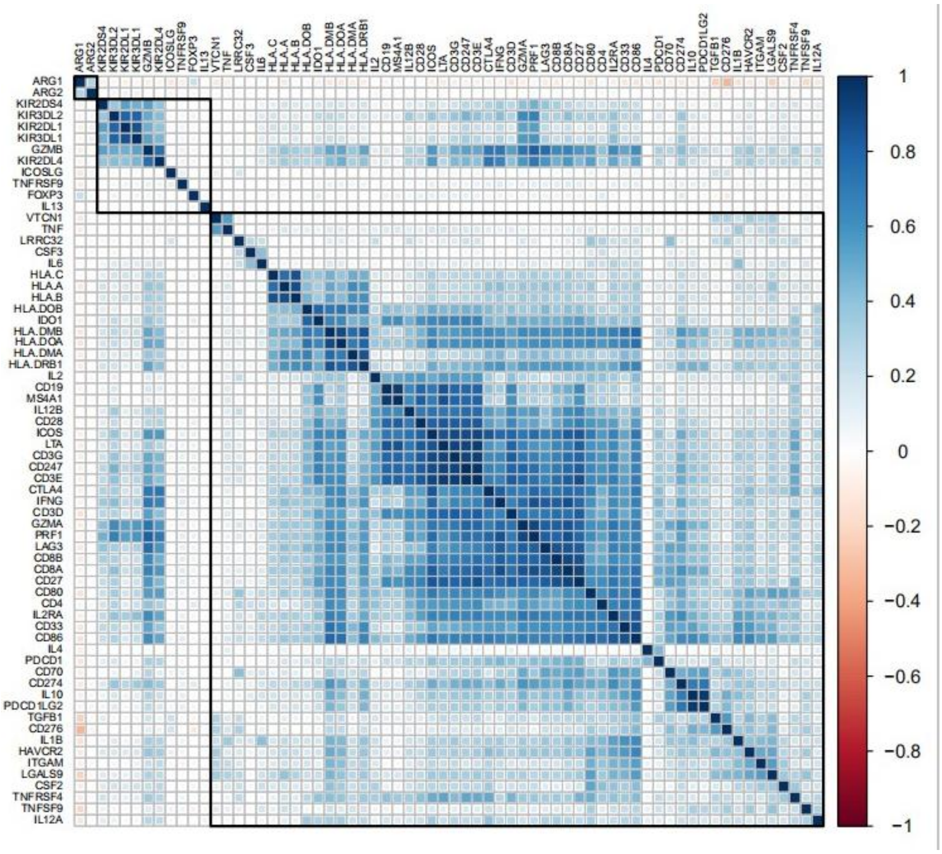

Supplementary Figure 3. Correlations between the 66 immune marker expression scores in all patients.

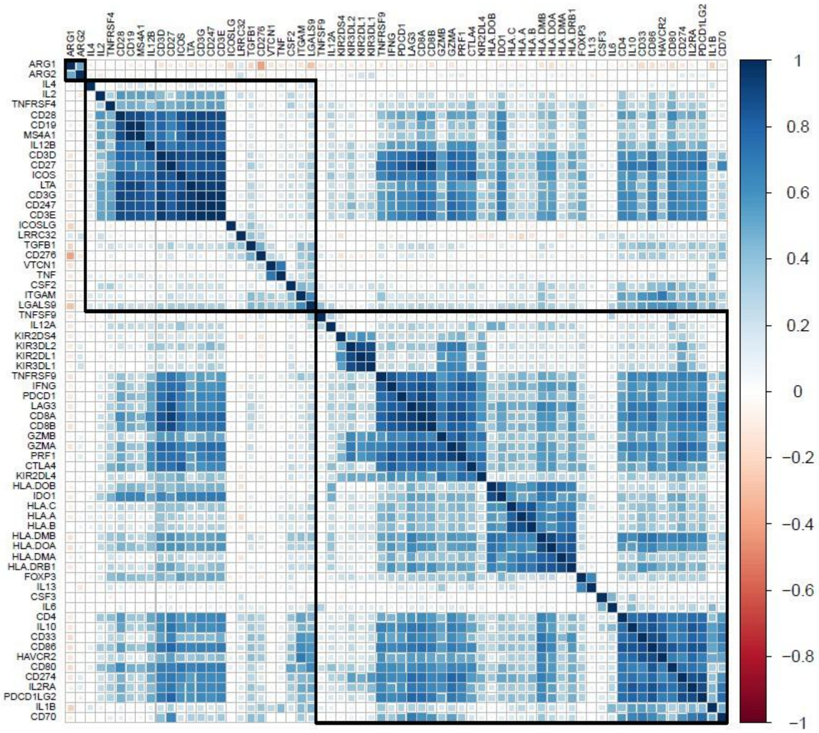

Supplementary Figure 4. Correlations between the 66 immune marker expression scores in patients of the high VEGF score subtype.

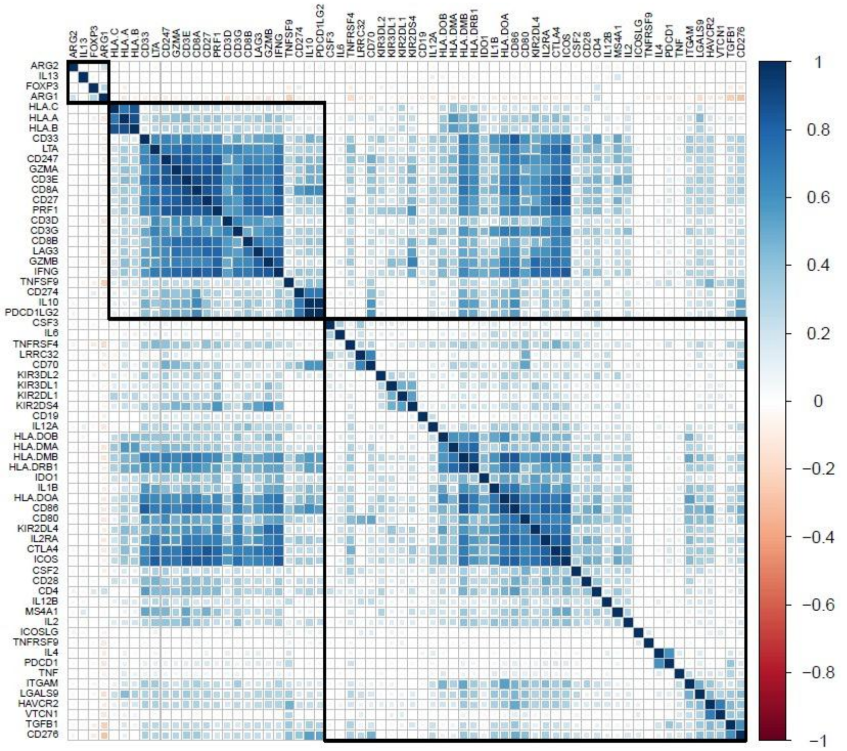

Supplementary Figure 5. Correlations between the 66 immune marker expression scores in patients of the low VEGF score subtypes.

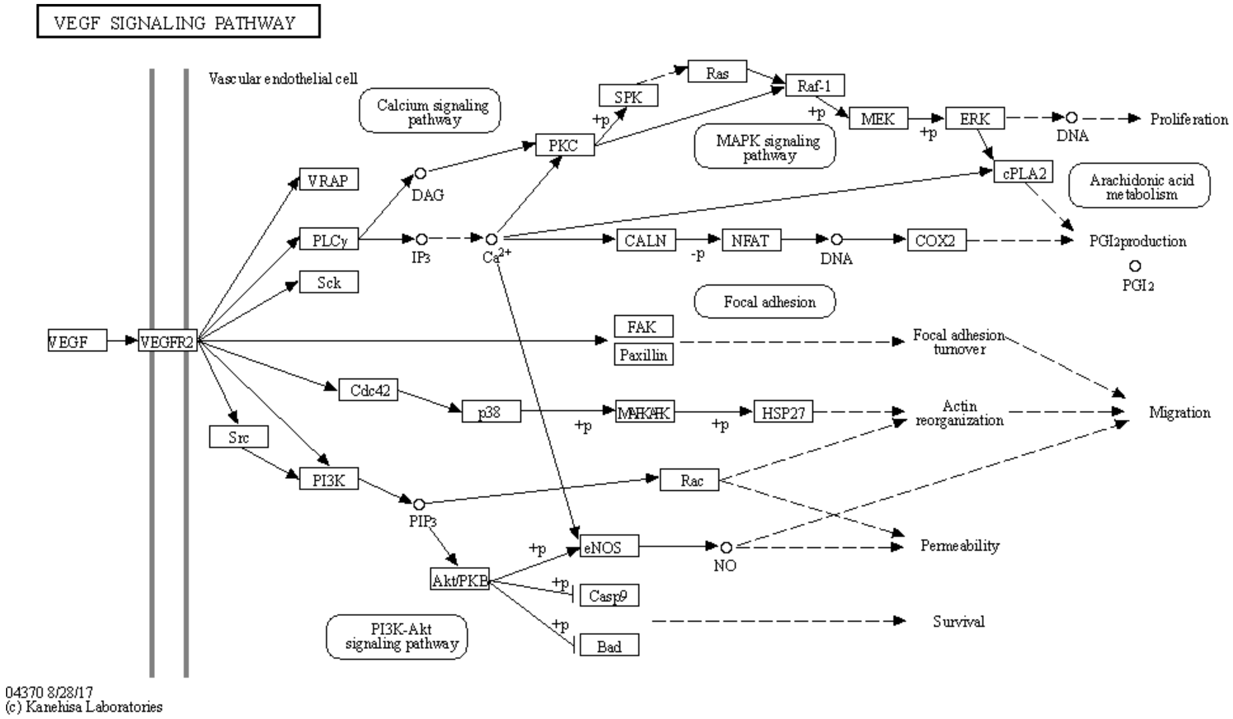

Supplementary Figure 6. The VEGF signaling pathway obtained from the KEGG pathway database (KEGG_VEGF_SIGNALING_PATHWAY).
